# A Systematic Review and Network Meta-Analysis on the Efficacy of Medications in the Treatment of Chronic Idiopathic Constipation in Japan

**DOI:** 10.1155/2021/5534687

**Published:** 2021-11-30

**Authors:** Atsushi Nakajima, Ayako Shoji, Kinya Kokubo, Ataru Igarashi

**Affiliations:** ^1^Gastroenterology and Hepatology, Yokohama City University Hospital, 3-9 Fukuura, Kanazawa-ku, Yokohama 236-0004, Japan; ^2^Medilead, Inc., Tokyo Opera City Tower, 24F 3-20-2 Nishishinjuku, Shinjuku-ku, Tokyo 163-1424, Japan; ^3^Department of Health Economics and Outcomes Research, Graduate School of Pharmaceutical Sciences, The University of Tokyo, 7-3-1, Hongo, Bunkyo-ku, Tokyo 113-0033, Japan; ^4^Faculty of International Politics and Economics, Nishogakusha University, 6-16, Sanbancho, Chiyoda-ku, Tokyo 102-8336, Japan; ^5^Unit of Public Health and Preventive Medicine, Yokohama City University School of Medicine, 3-9 Fukuura, Kanazawa-ku, Yokohama 236-0004, Japan

## Abstract

**Background:**

In the 2010s, medications with new mechanisms were introduced in Japan for the treatment of chronic idiopathic constipation (CIC). A few systematic reviews have compared medications' relative efficacy, but the reviews included studies on patients from various races, even though the mechanism of CIC is considered to differ between races. The aim of this study was to use a systematic review and network meta-analysis to compare the relative efficacy of these medications in Japanese patients.

**Methods:**

We conducted a meta-analysis and report it here according to the Preferred Reporting Items for Systematic Reviews and Meta-Analyses (PRISMA). We identified studies by searching MEDLINE (via the PubMed interface) and the Cochrane Library and ICHUSHI databases and included randomized clinical trials that compared medications for CIC with placebo in Japanese adults. Two reviewers independently screened and assessed articles, abstracted data, and assessed the risk of bias. We pooled data by random-effects meta-analyses and also performed a Bayesian network meta-analysis to indirectly compare data.

**Results:**

The present systematic review and meta-analyses included 1460 patients in 6 randomized clinical trials: 2 on linaclotide, 3 on elobixibat, 2 on lubiprostone, and 1 on lactulose. The results of direct comparisons showed that linaclotide, elobixibat, and lubiprostone were superior to placebo in the change of spontaneous bowel movements (SBMs) within 1 week: linaclotide, 1.95 (95% CI, 1.51-2.39); elobixibat, 5.69 (95% CI, 3.31-8.07); and lubiprostone, 2.41 (95% CI, 0.82-4.01). The Bayesian network meta-analysis showed consistent results. Elobixibat 10 mg was ranked first for the increase in SBMs and complete SBMs within 1 week and the time to first SBM. Lubiprostone 48 *μ*g was ranked first for the proportion of patients with SBM within 24 hours.

**Conclusion:**

Our direct and indirect meta-analyses revealed that the new CIC medications available in Japan have equal efficacy but that elobixibat and lubiprostone are highly likely to be more efficacious.

## 1. Introduction

Chronic idiopathic constipation (CIC) is highly prevalent in Japan [1, 2]. It impairs quality of life and causes a significant social and economic burden, and full recovery is difficult to achieve. Most patients with CIC are prescribed traditional medications that lack sufficient evidence of clinical efficacy and safety [3]. Recently, the long-term use and use of excessive doses of these traditional medications, especially in patients with comorbidities, have been associated with safety problems [4].

In the 2010s, the introduction of several medications with new mechanisms of action increased treatment options: in Japan, lubiprostone was introduced in 2012; linaclotide, in 2017; polyethylene glycol, in 2018; elobixibat, in 2018; and lactulose, in 2019. Randomized clinical trials (RCTs) of these medications found superior efficacy to placebo and thus raised expectations of improved disease outcomes in CIC [5–12]. The medications were expected to vary in efficacy because of their different mechanisms of action; however, every RCT was placebo-controlled and thus did not directly show whether any of the medications was superior to the others. Currently, in clinical practice, these medications are chosen according to their differences as presented in the respective package insert and not according to their validated efficacy.

Two other systematic reviews and meta-analyses indirectly compared the efficacy of the various medications used to treat CIC, but their results were inconsistent. For example, 1 of them indicated that all the medications have similar efficacy, although bisacodyl may be superior in improving spontaneous bowel movements (SBMs) [13], whereas the other suggested that prucalopride, a new medication not approved in Japan, is likely to be superior in increasing complete spontaneous bowel movements (CSBMs) [14]. Both of these papers included RCTs conducted in various countries, but neither focused on differences between countries.

Efficacy results of RCTs performed in Western countries and direct or indirect comparisons of these results are not necessarily generalizable to patients in Japan. Available CIC medications and their dosage forms differ between countries, e.g., prucalopride has not been approved in Japan. Furthermore, nutritional habits, which can affect bowel movements (for example, through differences in dietary fiber intake) [15, 16], are also likely to vary between countries. RCTs were performed of every new CIC medication now available in Japan, and their results were published, including endpoints relevant to the improvement in SBMs and CSBMs [5–12, 17]. Sufficient RCTs have been published to enable the relative efficacy of the drugs in Japanese patients to be evaluated. Therefore, the present study is aimed at indirectly comparing efficacy outcomes in CIC in Japanese patients by a systematic review and network meta-analysis of the results of these RCTs. The estimated efficacy in Japanese patients with CIC was expected to provide valuable information to help clinical practitioners in Japan and choose appropriate treatments for patients with CIC.

## 2. Materials and Methods

The protocol for this systematic review and network meta-analysis was registered with the International Prospective Register of Systematic Reviews (PROSPERO; CRD42020167825) [18], and the study is reported according to the Preferred Reporting Items for Systematic Reviews and Meta-Analyses (PRISMA) [19].

### 2.1. Study Criteria

Eligible studies had to meet the following criteria: (1) the study was a published RCT with at least 1-week follow-up; (2) the study population comprised Japanese adults (aged ≥ 18 years) with CIC diagnosed according to the Rome II, III, or IV criteria or variations of these; (3) the studied CIC medications were available in Japan at the time of the literature search for the present analysis, and the study compared their dosage forms with each other or with placebo; (4) the study evaluated relevant outcome measurements of bowel function, including SBMs; (5) the publication was written in English or Japanese; and (6) the study was published from 1 January 2010 to 31 December 2019 and included medications that were approved in the 2010s in Japan. Studies were excluded if they were not original research, were observational studies, were post hoc analyses, were focused on patients with chronic constipation as a complication or side effect of other diseases, or were conducted in hospitals or medical centers outside Japan. If we found 2 or more publications on 1 RCT (e.g., in both English and Japanese), we included the most comprehensive report.

### 2.2. Search Strategy

To identify potentially relevant articles, in March 2020, we conducted a systematic search in MEDLINE (via the PubMed interface), the Cochrane Library database, and the ICHUSHI database (a database of articles written in Japanese). The search used the following text words and database-specific index terms combined with operators (“AND” and “OR”): (constipation[MeSH] OR “chronic constipation” OR “chronic idiopathic constipation”) AND (AJG555 OR A3309 OR DB1248 OR elobixibat OR Goofice OR “IBAT inhibitor” OR ASP0456 OR linaclotide OR Linzess OR constella OR lubiprostone OR Amitiza OR Macrogol OR Movicol OR “polyethylene glycol” OR “magnesium oxide” OR “SK-1202” OR “crystalline lactulose preparation” OR “crystallized lactulose preparation”). The search was restricted to studies published in the period 2010 to 2019. The detailed search terms and retrieval records are shown in Supplementary Tables 1 and 2. We also searched in registration systems of clinical trials and package inserts of the approved CIC medications to confirm that they did not refer to any other reports that had not been published.

First, 2 reviewers (AS and KK) independently reviewed the titles and abstracts and excluded studies that did not meet the inclusion criteria. The full texts of the remaining articles were then obtained, and the 2 reviewers independently evaluated whether the studies met the inclusion criteria. Discrepancies between the 2 reviewers were resolved through discussions with a third reviewer (AI).

### 2.3. Study Outcomes

Data were extracted by AS and reviewed for accuracy by AI. The following information was extracted from each eligible article: first author, study year, number of cases, number of controls, age, number of women and men, medication studied, dosage form of medication, number of measurement days, follow-up period, proportion of patients with irritable bowel syndrome constipation, and variables in 7 relevant domains (see below) for the assessment of the risk of bias. Outcome data were also extracted, including the following: mean change in weekly SBMs, mean change in weekly CSBMs, mean time to first SBM after baseline, and proportion of patients with SBM within 24 hours after the first administration of the medication. If SDs were not specified for the means, we extracted measures that enabled us to estimate SD (e.g., SEMs or CIs). If outcome measurements were reported at multiple times, we used the data from the time closest to that most often reported across the RCTs. If numerical data were not specified in the text or tables, we obtained them from the figures.

### 2.4. Quality Assessment

Two reviewers (AI and AS) independently assessed the quality of the included studies with the Cochrane risk of bias tool [20]. The assessment considered the following domains: generation of random sequences and concealment of allocation (selection bias), blinding of participants and personnel (performance bias), incomplete outcome assessment (attrition bias), selective reporting (reporting bias), and other sources of bias. Each of these domains was categorized as being a low (+), unclear (?), or high (-) risk of bias according to the recommendations outlined in the Cochrane Handbook for Systematic Reviews of Interventions (version 5.2) [20]. Disagreements were resolved through discussion.

### 2.5. Statistical Analysis

Data were abstracted and analyzed by R (version 3.6.0, R Foundation for Statistical Computing). For traditional meta-analyses, we used “metafor” (version 2.0-0), and for Bayesian network meta-analyses, the “gemtc” package (version 0.8-2) and JAGS (version 4.3.0, MRC Biostatistics Unit, Cambridge, UK). We calculated mean differences (MDs) for mean values and odds ratios (ORs) for proportions and reported them with 95% CIs or 95% credible intervals (CrIs); statistical significance was defined as *P* < 0.05. All statistical tests were 2-sided. Publication bias was assessed with funnel plots, but no statistical test of funnel plot asymmetry was used because at least 10 studies are required to detect true asymmetry [20].

We first conducted traditional pairwise meta-analyses for every treatment (i.e., direct comparisons) with the DerSimonian and Laird random-effects model (“metafor” package on R). We assessed statistical heterogeneity with the *I*^2^ statistic, which describes the proportion of the variation that is related to heterogeneity rather than chance, and the *Q* test; an *I*^2^ greater than 50% or a *P* value less than 0.05 was considered to indicate substantial heterogeneity. Sources of heterogeneity between studies were investigated by performing subgroup analyses of study characteristics.

We also conducted a Bayesian hierarchical network meta-analysis with a random-effects model that used noninformative priors and a Markov chain Monte Carlo (MCMC) simulation (“gemtc” package, which recalls JAGS in R for MCMC sampling). We used 4 parallel chains and ran 20000 simulations to obtain model parameters after 5000 burn-in samples for each chain. To check convergence, we used the Gelman-Rubin diagnostic and trace plots. The rank probabilities were calculated to obtain the hierarchy of each treatment. In addition to the comparisons between every dosage form of every treatment, we conducted network meta-analyses with the typical doses most commonly administered in clinical practice in Japan.

When conducting network meta-analyses, one must assume consistency of the network to be analyzed and the absence of conflicts between the results of direct and indirect comparisons. Therefore, we assessed the consistency between the direct and indirect comparisons by comparing the pooled MDs and ORs from the traditional pairwise meta-analyses and network meta-analyses. We could not implement the node-splitting method because the comparator was placebo in all eligible studies, and no loop connected the 3 arms.

## 3. Results

### 3.1. Search Results

**Figure 1:**
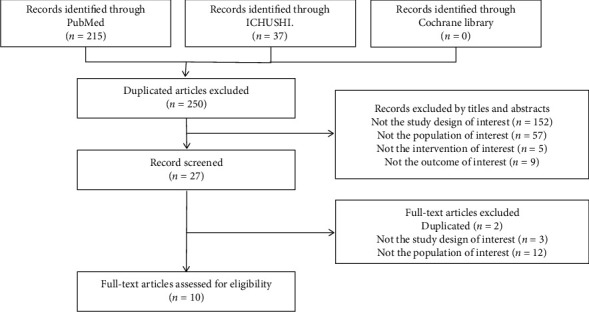
The flow diagram of the literature selection process.

A flow diagram of the literature selection process is shown in Figure 1. The initial search identified 252 articles, 250 of which were deemed potentially relevant after removal of duplicate or irrelevant references (Figure 1). No articles on Japanese patients with CIC were found in the Cochrane Library database. After reviewing the unique titles and abstracts, we selected 27 articles for further full-text review. Of these 27 articles, 17 were excluded: 9 did not include Japanese patients, 3 were not RCTs, 3 included patients with diseases or treatments that could have caused CIC, and 2 reported the same results as other articles. Therefore, 10 articles met the inclusion and exclusion criteria and were available for analysis.

The study characteristics are summarized in Table 1. All included studies were RCTs conducted at medical centers in Japan and published in the period 2010 to 2019 (Table 1). Most of the studies included patients with irritable bowel syndrome constipation. Of the 10 eligible studies, 2 RCTs on magnesium oxide [21] and polyethylene glycol [17] were omitted from the main analyses: magnesium oxide is used as a first-line treatment option for patients with CIC, whereas the other medications are used for patients who do not respond to first-line treatment, and polyethylene glycol can be titrated when symptoms improve, whereas the other medications are used at fixed doses. Ultimately, 8 RCTs on 4 medications were included in the present systematic review and meta-analyses: 2 on linaclotide [7, 8], 3 on elobixibat [9–11], 2 on lubiprostone [5, 6], and 1 on lactulose [12] (Table 1). A total of 1460 patients were enrolled in the 8 studies, all of which used the Rome III criteria for diagnosing CIC.

**Figure 2:**
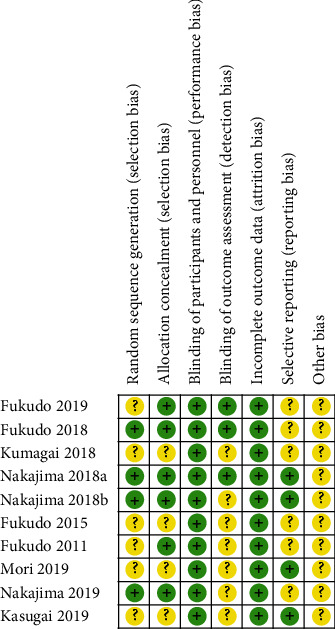
Risk of bias summary of reviewers' judgements for each risk of bias item for selected studies.

The results of the quality assessment of the 8 studies according to the Cochrane risk of bias tool are shown in Figure 2. Downgrading of quality because of an “unclear risk of bias” was based on an insufficient or incomplete description of random sequence generation, blinding, allocation concealment, or missing data management.

The extracted endpoints of the 8 studies are summarized in Table 2. All 8 studies reported efficacy according to the change of SBMs in the first week. Seven studies reported the proportion of patients with SBM within 24 hours [5–7, 9–12]; 5, the time to first SBM [6, 7, 10–12]; and 4, the change in CSBMs in the first week [7, 8, 10, 11]. No studies reported adjusted effects.

### 3.2. Meta-Analyses

#### 3.2.1. Direct Comparison

**Figure 3:**
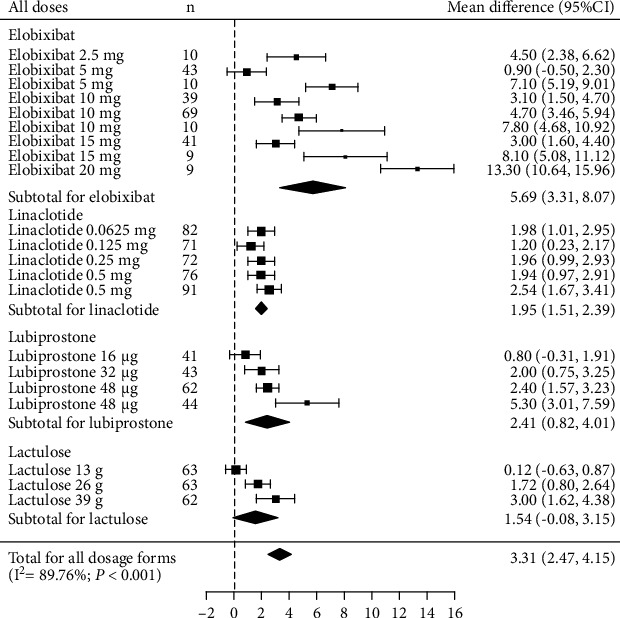
Forrest plot of random-effects meta-analysis results in change in weekly SBMs compared between all doses. SBM: spontaneous bowel movement.

The results of the direct comparisons are shown in Figure 3. The dosage forms of medications that showed more than 7.0 MDs in SMBs within 1 week were extracted from a phase 1 trial of elobixibat (Figure 3). Three of the 4 medications showed a significant increase in SBMs within 1 week compared with placebo: the MD from placebo was 5.69 (95% CI, 3.31-8.07) for elobixibat, 1.95 (95% CI, 1.51-2.39) for linaclotide, and 2.41 (95% CI, 0.82-4.01) for lubiprostone. These wide and overlapping 95% CIs indicated similar efficacy among the 3 medications. Lactulose did not show a significant difference compared with placebo. The studies showed significant heterogeneity (*I*^2^ = 89.76%; *P* < 0.001).

Levels of heterogeneity were not substantially lower when studies were stratified by their characteristics as follows: studies that included patients with IBS-C (6 studies), *I*^2^ = 92.03%, *P* < 0.001 [5, 6, 9–12]; studies that did not include patients with IBS-C (2 studies), *I*^2^ = 1.21%, *P* = 0.399 [7, 8]; studies with a mean baseline age above 40 years (6 studies), *I*^2^ = 78.37%, *P* < 0.001 [6–8, 10–12]; studies that assessed linaclotide or lubiprostone (4 studies), *I*^2^ = 54.14%, *P* = 0.026 [5–8]; studies that assessed linaclotide, lubiprostone, or SK1202 (5 studies), *I*^2^ = 72.86%, *P* < 0.001 [5–8, 12]; studies that assessed elobixibat (3 studies), *I*^2^ = 91.27%, *P* < 0.001 [9–11]; phase II or III studies (7 studies), *I*^2^ = 77.87%, *P* < 0.001 [5–8, 10–12]; phase III studies (3 studies), *I*^2^ = 80.29%, *P* = 0.006 [6, 7, 10]; and studies published after 2018 (6 studies), *I*^2^ = 91.19%, *P* < 0.001 [7–12]. The funnel plot appeared to be asymmetric (Supplementary Figure 1).

**Figure 4:**
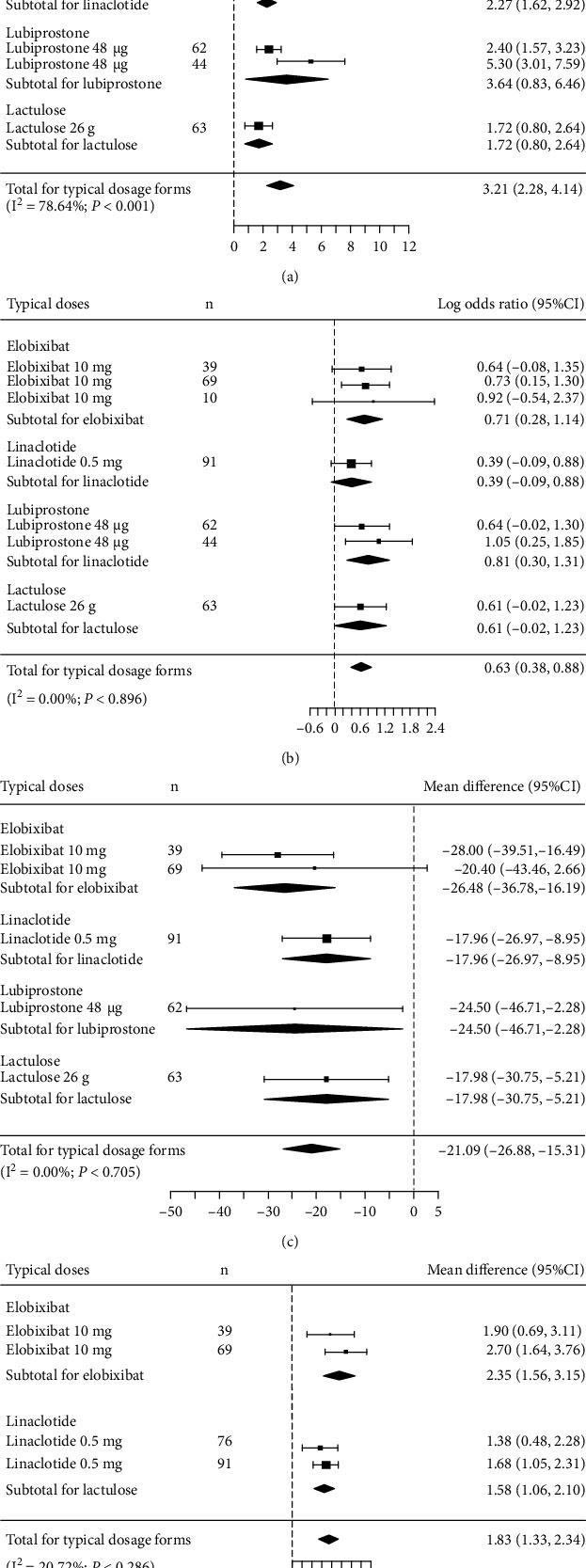
Forrest plot of random-effects meta-analysis results: (a) change in weekly SBMs compared between typical doses; (b) proportion of patients with SBM within 24 hours compared between typical doses; (c) time to first SBMs compared between typical doses; (d) change in weekly CSBMs compared between all doses. CSBM: complete spontaneous bowel movement; SBM: spontaneous bowel movement.

In the direct comparisons of the typical dose of each medication, all 4 medications showed a significant increase in SBMs within 1 week compared with placebo. The MDs were as follows: elobixibat 10 mg, 4.88 (95% CI, 2.53-7.24); linaclotide 0.5 mg, 2.27 (95% CI, 1.62-2.92); lubiprostone 48 *μ*g, 3.64 (95% CI, 0.83-6.46); and lactulose 26 g, 1.72 (95% CI, 0.80-2.64) (Figure 4(a)). The 95% CIs overlapped, indicating similar efficacy. Heterogeneity among the studies was significant (*I*^2^ = 78.64%; *P* < 0.001). Time to first SBM was significantly lower for all 4 medications than for placebo; the MDs were as follows: elobixibat 10 mg, -26.48 (95% CI, -36.78 to -16.19); linaclotide 0.5 mg, -17.96 (95% CI, -26.97 to -8.95); lubiprostone 48 *μ*g, -24.50 (95% CI, -46.71 to -2.28); and lactulose 26 g, -17.98 (95% CI, -30.75 to -5.21) (Figure 4(c)). The comparisons of the proportion of patients with SBM within 24 hours and the change in CSBMs within 1 week showed significant increases for 3 medications (2 as ORs and 2 as MDs): elobixibat 10 mg, OR = 0.71 (95% CI, 0.28-1.14) and MD = 2.35 (95% CI, 1.56-3.15); linaclotide 0.5 mg, MD = 1.58 (95% CI, 1.06-2.10); and lubiprostone 48 *μ*g, OR = 0.81 (95% CI, 0.30-1.31) (Figures 4(b) and 4(d)). We found no significant heterogeneity in the direct comparisons of time to first SBM (*I*^2^ = 0.00%; *P* = 0.705), proportion of patients with SBM within 24 hours (*I*^2^ = 0.00%; *P* = 0.896), or change in CSBMs within 1 week (*I*^2^ = 20.72%; *P* = 0.286).

#### 3.2.2. Indirect Comparison

**Figure 5:**
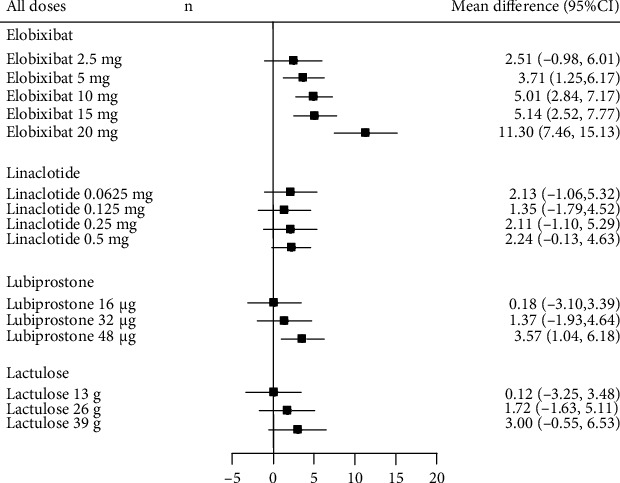
Forrest plot of Bayesian network meta-analysis results of the change in weekly SBMs compared between all doses.

The results of the Bayesian network meta-analysis of the 8 studies (Figure 5) were consistent with the results of the direct comparisons (Figure 3), i.e., all dosage forms showed a higher increase in SBMs within 1 week compared with placebo, although most of the increases were not statistically significant. In the analyses of the typical dose of each medication, 3 of the 4 medications showed significant increases in SBMs within 1 week compared with placebo, but the medications did not differ significantly from each other in the results of the Bayesian network meta-analysis (Table 3(a)). The results for the other endpoints were similar (Tables 3(b)–3(d)).

**Figure 6:**
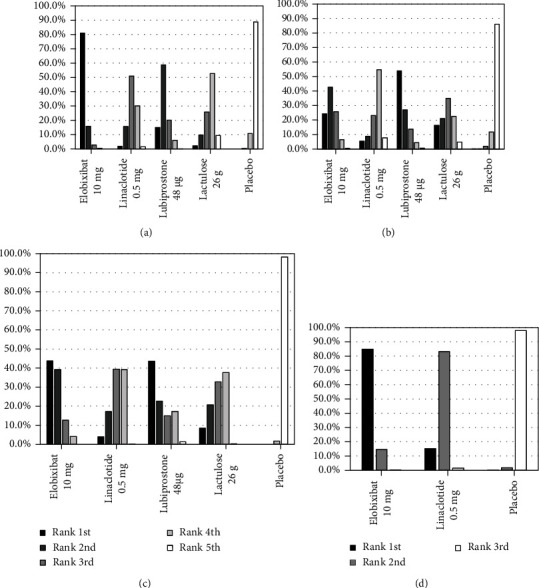
Rank probabilities of typical doses of subject medications: (a) change in weekly SBMs; (b) proportion of patients with SBM within 24 hours; (c) time to first SBMs; (d) change in weekly CSBMs. CSBM: complete spontaneous bowel movement; SBM: spontaneous bowel movement.

The results of the treatment rank analyses of the typical dose of each medication (Figure 6) were consistent with the estimated treatment effect sizes in the direct and indirect comparisons. Elobixibat 10 mg was ranked first for 3 endpoints, with a rank probability of 81.0% for the increase in SBMs within 1 week (Figure 6(a)), 43.8% for the time to first SBM (Figure 6(c)), and 84.8% for the increase in CSBMs within 1 week (Figure 6(d)). Lubiprostone 48 *μ*g was ranked first for the proportion of patients with SBM within 24 hours (Figure 6(b)). Linaclotide 0.5 mg or lactulose 26 g was ranked last among the 4 medications.

## 4. Discussion

The present study combined the results of RCTs on 4 new medications available to treat CIC in Japanese patients. Our direct and indirect comparisons showed that elobixibat and lubiprostone significantly improved bowel movements compared with placebo and that all 4 medications had similar efficacy in all other endpoints. The likelihood of being ranked first was higher for elobixibat and lubiprostone. Currently, physicians choose among the 4 medications by considering the differences described in the package inserts: lubiprostone is contraindicated for pregnant women; linaclotide is reported to be associated with severe diarrhea; and elobixibat has the potential to have low efficacy in patients with severe hepatic disorders and should be used with care in these patients [22]. However, our results suggest that these medications can be selected according to their efficacy.

As mentioned in the “Introduction,” 2 other systematic reviews and network meta-analyses examined CIC treatments in patients of various races, including Japanese patients, and they had different findings. One of them, published in 2016, evaluated the 4 medications included in the present analyses, as well as traditional medications and other medications approved only in Western countries [13]. It included studies whose endpoints were not in line with those currently recommended by the FDA and found that all medications, in particular linaclotide, were superior to placebo and showed equal efficacy in improving bowel movements. This finding was in line with our results. The other meta-analysis, published in 2019, focused on the failure to achieve an increase of 1 or more CSBMs as an endpoint and showed significant differences between the CIC medications studied, indicating that the medications did not necessarily have equal efficacy regarding some treatment goals [14]. The latter meta-analysis also included RCTs that were prematurely terminated, whereas the former meta-analysis included only completed RCTs, similar to our study. Both studies included results of studies in patients with CIC in Western countries and focused on higher doses than those appropriate in Japanese patients. The difference in the findings of these 2 studies is considered to be associated with the difference in the eligibility criteria and endpoints. The present study included only completed RCTs, similar to one of the previous studies [13], but it also limited eligible studies to those conducted in Japanese populations with CIC; it revealed that typical doses of elobixibat and lubiprostone are superior to placebo in some clinically important endpoints. In previous studies, prucalopride showed significantly higher efficacy than other medications, but it has not been approved in Japan.

In the RCTs included in the present study, the patients were included on the basis of common inclusion criteria used globally (based on Rome II, III, or IV) and thus might not be representative of Japanese CIC populations treated in real-world clinical settings, for example, because the relevant Japanese guideline includes many diagnostic components based not only on the Rome criteria but also on several guidelines and results of meta-analyses [3]. Issues of importance in CIC are not only bowel movements, which were the primary and secondary endpoints of the RCTs, but also social aspects, such as quality of life [23]. However, the results of the present meta-analyses reflect only the efficacy in improving bowel movements. Another recent meta-analysis indicated that patients in Europe tended to have a longer stool transit time in the bowel than patients in the United States and Asia [24]. Therefore, further clinical endpoints reflecting real-world clinical conditions and issues other than bowel movements need to be established for clinical trials and routine clinical care. The evaluation of medications on the basis of such endpoints has become more important as more treatment options have become available.

This study has some limitations. First, we found significant heterogeneity between the studies, which was likely to be associated with the asymmetry seen in the funnel plots; however, in the subgroup analyses, we were unable to find any clinical characteristics of studies that contributed to this significant heterogeneity. Because our study focused on the Japanese CIC population, the number of eligible studies and patients was limited, and the 95% CIs were wider than those of recent studies that included RCTs with large numbers of patients, such as international collaborative studies. As the number of Japanese and head-to-head trials increases, differences may be detected between medications, allowing the accuracy of our results to be tested. Second, all analyses were conducted with 4 endpoints, but the target endpoints differed between the eligible studies, and only 4 studies of 2 medications compared the increase in CSBMs in the first week. The various rank probabilities of the endpoints in our study indicated that the advantage of each CIC medication has not yet been assessed adequately enough to inform appropriate treatment options in routine clinical practice. Future clinical trials need to include more comprehensive endpoints.

## 5. Conclusions

Our direct and indirect meta-analyses revealed that the new CIC medications available in Japan have equal efficacy but that elobixibat and lubiprostone are highly likely to be more efficacious. Future studies may consider evaluating the efficacy of CIC medications with other clinical indexes, such as the Bristol stool form score or the Patient Assessment of Constipation Quality of Life Questionnaire [3].

## Figures and Tables

**Table 1:** All eligible studies.

Study ID	Loc	Diagnosis	IBS-C	*N* of total patients	Age (mean ± SD)	*P* of females	Intervention	Trial stage	Ref no.	MA
Fukudo 2019	Japan	<3 SBMs per week, Rome III diagnostic criteria for functional constipation	Not included	181	42.7 ± 11.9	82.3%	Linaclotide 0.5 mg	III	7	Included
Fukudo 2018	Japan	<3 SBMs per week, Rome III diagnostic criteria for functional constipation	Not included	382	41.6 ± 11.4	83.2%	Linaclotide 0.0625 mg Linaclotide 0.125 mg Linaclotide 0.25 mg Linaclotide 0.5 mg	II	8	Included
Kumagai 2018	Japan	<3 SBMs per week, Rome III diagnostic criteria for functional constipation	Included	58	35.4 ± 10.8	Not reported	Elobixibat 2.5 mg Elobixibat 5 mg Elobixibat 10 mg Elobixibat 15 mg Elobixibat 20 mg	I	9	Included
Nakajima 2018a	Japan	Rome III diagnostic criteria for functional constipation	Included	132	43.4 ± 13.3	82.6%	Elobixibat 10 mg	III	10	Included
Nakajima 2018b	Japan	Rome III diagnostic criteria for functional constipation	Included	163	44.6 ± 12.8	87.7%	Elobixibat 5 mg Elobixibat 10 mg Elobixibat 15 mg	II	11	Included
Fukudo 2015	Japan	<3 SBMs per week, Rome III diagnostic criteria for functional constipation	Included	124	42.1 ± 15.3	87.9%	Lubiprostone 48 mg	III	6	Included
Fukudo 2011	Japan	<3 SBMs per week, Rome III diagnostic criteria for functional constipation	Included	170	39.5 ± 11.7	90.6%	Lubiprostone 16 *μ*g Lubiprostone 32 *μ*g Lubiprostone 48 *μ*g	II	5	Included
Mori 2019	Japan	Rome IV diagnostic criteria for functional constipation	Included	33	40.9 ± 12.8	100.0%	Magnesium oxide	—	21	
Nakajima 2019	Japan	<3 SBMs per week, Rome III diagnostic criteria for functional constipation	Included	156	43.2 ± 12.2	85.6%	Polyethylene glycol 3350 plus electrolytes	III	17	
Kasugai 2019	Japan	<3 SBMs per week, Rome III diagnostic criteria for functional constipation	Included	250	41.9 ± 11.9	84.0%	Lactulose 13 g/day Lactulose 26 g/day Lactulose 39 g/day	II	12	Included

IBS-C: irritable bowel syndrome with constipation; Loc: location; MA: meta-analysis; *N*: number; *P*: proportion; Ref no.: reference number; SBM: spontaneous bowel movement.

**Table 2:** Extracted information for meta-analyses.

Study ID, intervention, trial stage	Treatment	*N*	Change in weekly SBMs		SBMs within 24 hrs	Time to first SBMs		Change in weekly CSBMs	
			Mean	SE^∗^	%	Mean	SE^∗^	Mean	SE^∗^
Fukudo 2019, linaclotide, III [7]	Placebo	88	1.48	0.32	48.30	24.67	3.15	0.78	0.23
	Linaclotide 5 mg	91	4.02	0.31	72.80	6.71	3.35	2.46	0.23

Fukudo 2018, linaclotide, II [8]	Placebo	80	1.91	0.35^†^	—	—	—	1.10	0.30^†^
	Linaclotide 0.0625 mg	82	3.89	0.35^†^	—	—	—	2.16	0.30^†^
	Linaclotide 0.125 mg	71	3.11	0.35^†^	—	—	—	2.23	0.30^†^
	Linaclotide 0.25 mg	72	3.87	0.35^†^	—	—	—	2.53	0.35^†^
	Linaclotide 0.5 mg	76	3.85	0.35^†^	—	—	—	2.48	0.35^†^

Kumagai 2018, elobixibat, I [9]	Placebo	10	1.40	0.57^†^	40.00	—	—	—	—
	Elobixibat 2.5 mg	10	5.90	0.92^†^	100.00	—	—	—	—
	Elobixibat 5 mg	10	8.50	0.79^†^	100.00	—	—	—	—
	Elobixibat 10 mg	10	9.20	1.49^†^	100.00	—	—	—	—
	Elobixibat 15 mg	9	9.50	1.43^†^	100.00	—	—	—	—
	Elobixibat 20 mg	9	14.70	1.23^†^	88.90	—	—	—	—

Nakajima 2018a, elobixibat, III [10]	Placebo	63	1.70	0.20	41.00	25.50	11.63	0.60^†^	0.20^†^
	Elobixibat 10 mg	69	6.40	0.60	86.00	5.10	1.80	3.30^†^	0.50^†^

Nakajima 2018b, elobixibat, II [11]	Placebo	40	2.60	0.46^†^	48.00	36.20	5.72^†^	1.50	0.22^†^
	Elobixibat 5 mg	43	3.50	0.55^†^	61.00	19.90	3.03^†^	2.00	0.32^†^
	Elobixibat 10 mg	39	5.70	0.67^†^	90.00	8.20	1.31^†^	3.40	0.58^†^
	Elobixibat 15 mg	41	5.60	0.55^†^	93.00	8.50	1.33^†^	3.80	0.55^†^

Fukudo 2015, lubiprostone, III [6]	Placebo	62	1.26	0.23	30.60	48.03	10.83^†^	—	—
	Lubiprostone 48 *μ*g	62	3.66	0.36	58.10	23.53	3.33^†^	—	—

Fukudo 2011, lubiprostone, II [5]	Placebo	42	1.50	0.40	26.20	—	—	—	—
	Lubiprostone 16 *μ*g	41	2.30	0.40	53.70	—	—	—	—
	Lubiprostone 32 *μ*g	43	3.50	0.50	53.50	—	—	—	—
	Lubiprostone 48 *μ*g	44	6.80	1.10	75.00	—	—	—	—

Kasugai 2019, lactulose, II [12]	Placebo	62	2.05	0.28^†^	35.50	27.98	5.92	—	—
	Lactulose 13 g	63	2.17	0.26^†^	47.60	24.50	3.36	—	—
	Lactulose 26 g	63	3.77	0.38^†^	65.10	10.00	2.73	—	—
	Lactulose 39 g	62	5.05	0.65^†^	67.70	10.33	4.28	—	—

CI: confidence interval; CSBM: complete spontaneous bowel movement; hrs: hours; SBM: spontaneous bowel movement. ^∗^If SEs were not reported, they were calculated from SDs or 95% CIs. ^†^Obtained from figures.

**Table:** (a) Change in weekly SBMs (mean difference and corresponding 95% Crls)

*Elobixibat 10 mg*				
2.406 (-0.166, 5.322)	*Linaclotide 0.5 mg*			
1.359 (-1.611, 4.134)	-1.047 (-4.286, 1.648)	*Lubiprostone 48 μg*		
2.952 (-0.232, 6.544)	0.547 (-2.895, 4.025)	1.579 (-1.690, 5.463)	*Lactulose 26 g*	
4.663 (2.932, 6.696)	2.249 (0.226, 4.241)	3.306 (1.353, 5.752)	1.704 (-1.142, 4.503)	*Placebo*

**Table:** (b) Proportion of patients with SBM within 24 hours (relative risk and corresponding 95% Crls)

*Elobixibat 10 mg*				
1.374 (0.529, 3.839)	*Linaclotide 0.5 mg*			
0.889 (0.390, 2.035)	0.645 (0.218, 1.800)	*Lubiprostone 48 μg*		
1.149 (0.433, 3.302)	0.836 (0.247, 2.829)	1.295 (0.453, 3.910)	*Lactulose 26 g*	
2.051 (1.277, 3.579)	1.493 (0.640, 3.516)	2.317 (1.259, 4.542)	1.789 (0.749, 4.308)	*Placebo*

**Table:** (c) Time to first SBM (mean difference and corresponding 95% Crls)

*Elobixibat 10 mg*				
-8.362 (-22.320, 5.561)	*Linaclotide 0.5 mg*			
-0.735 (-25.167, 22.572)	7.620 (-17.334, 31.086)	*Lubiprostone 48 μg*		
-8.072 (-24.794, 9.007)	0.514 (-16.070, 16.286)	-7.219 (-32.774, 18.158)	*Lactulose 26 g*	
-26.438 (-36.588, -15.795)	-17.961 (-27.797, -8.897)	-25.481 (-47.119, -3.493)	-18.403 (-31.464, -5.301)	*Placebo*

**Table:** (d) Changes in weekly CSBMs (mean difference and corresponding 95% Crls)

*Elobixibat 10 mg*		
0.779 (-1.194, 2.692)	*Linaclotide 0.5 mg*	
2.336 (0.885, 3.716)	1.552 (0.230, 2.841)	*Placebo*

Crl: credible interval; CSBM: complete spontaneous bowel movement; SBM: spontaneous bowel movement.

## Data Availability

This is a systematic review, and all information used for analyses was presented in the article.

## Supplementary Materials

Supplementary Materials The supplementary files contain the following information: the detailed search strategies, the funnel plots, and the PRISMA checklist.
