# Supplementary material for: A Systematic Review and Network Meta-Analysis on the Efficacy of Medications in the Treatment of Chronic Idiopathic Constipation in Japan
Source: Gastroenterol Res Pract. 2021 Nov 30;2021:5534687. doi: 10.1155/2021/5534687 (PMC8651382; doi:10.1155/2021/5534687)
Supplement: Supplementary Materials — The supplementary files contain the following information: the detailed search strategies, the funnel plots, and the PRISMA checklist. [file 5534687.f1.zip › Supplementary_20210130_cic_nma_AtsushiNakajima_rev28Sep2021.docx]

Supplementary Table 1: Search Strategy in MEDLINE via PubMed.

| # | Searches | Results |
| --- | --- | --- |
| 1 | Constipation[MeSH Terms] | 13,816 |
| 2 | “chronic constipation”[tw] OR "chronic idiopathic constipation"[tw] | 3,095 |
| 3 | #1 OR #2 | 14,831 |
| 4 | AJG555[tiab] OR A3309[tiab] OR DB1248[tiab] OR elobixibat[tiab] OR Goofice[tiab] OR ”IBAT inhibitor”[tiab] OR ASP0456[tiab] OR linaclotide[tiab] OR Linzess[tiab] OR constella[tiab] OR lubiprostone[tiab] OR Amitiza[tiab] OR Macrogol[tiab] OR MOVICOL[tiab] OR "Polyethylene Glycol"[tiab] OR "Magnesium oxide"[tiab] OR "SK?1202"[tiab] OR "crystalline lactulose preparation"[tiab] OR "crystallized lactulose preparation"[tiab] | 26,855 |
| 5 | (randomized controlled trial [pt] OR controlled clinical trial [pt] OR randomized [tiab] OR placebo [tiab] OR clinical trials as topic [mesh: noexp] OR randomly [tiab] OR trial [ti]) NOT (animals [mh] NOT humans [mh]) | 1,181,589 |
| 6 | non-Randomized Controlled Trials as Topic[mh] OR Controlled clinical trial [pt] OR Controlled Clinical Trials as Topic [mh] OR comparative study[pt] OR Clinical trial[pt] OR clinical trial [mh] OR Clinical trials as topic[mh] OR cross-over studies [mh] | 2,746,914 |
| 7 | meta-analysis [pt] OR meta-analysis [mh] OR “meta-analysis” [All] or (systematic[sb] AND review[pt]) OR “systematic review” [All] | 275,225 |
| 8 | #5 OR #6 OR #7 | 3,258,407 |
| 9 | #3 AND #4 AND #8 | 317 |
| 10 | (#3 AND #4 AND #8) Filters: Publication date from 1 January 2010 to 31 December 2019 | 215 |

Supplementary Table 2: Search Strategy in ICHUSHI^†^.

| 1 | "chronic constipation" or "chronic idiopathic constipation" | 1,388 |
| --- | --- | --- |
| 2 | "AJG555” OR ”A3309” OR ”DB1248” OR ”elobixibat” OR ”Goofice” OR ”IBAT inhibitor” OR ”ASP0456” OR ”linaclotide” OR ”Linzess” OR ”constella” OR ”lubiprostone” OR ”Amitiza" OR "Macrogol" OR "MOVICOL" OR "Polyethylene Glycol" OR "Magnesium oxide" OR "SK?1202" OR "crystalline lactulose preparation" OR "crystallized lactulose preparation"" OR "LAGNOS" | 19,771 |
| 3 | "Clinical trials" OR "Clinical trial" OR "Clinical study" OR "Clinical studies" OR "Clinical research" OR "Comparative study" OR "Comparative studies" OR "Comparative research" OR "comparison study" OR "comparison research" OR "Meta-analysis" OR "Systematic Review" | 475,880 |
| 4 | #1 AND #2 AND #3 | 44 |
| 5 | #4 AND (PDAT=2010/1/1:2019/12/31) | 37 |

† All words were translated in Japanese and added.


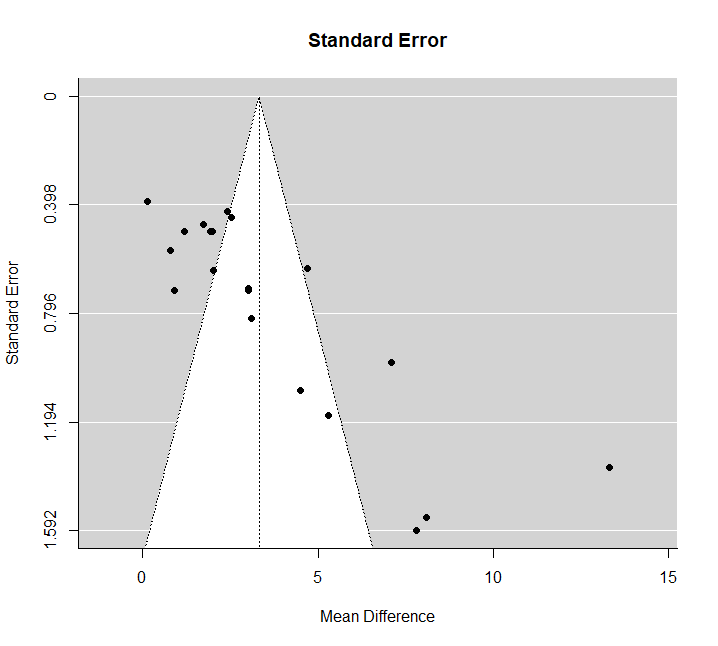


Supplementary Figure 1: The funnel plot on change in weekly spontaneous bowel movements.
